# Supplementary material for: Estimation of cost efficiency of fattening pigs, sows, and piglets using SFA approach analysis: Evidence from China
Source: PLoS One. 2021 Dec 13;16(12):e0261240. doi: 10.1371/journal.pone.0261240 (PMC8668113; doi:10.1371/journal.pone.0261240)
Supplement: Attachment — Submitted filename: Response to Reviewers.docx [file pone.0261240.s004.docx]

Response to Reviewers

Dear editors and reviewers,

Thank you for your valuable feedback of our manuscript entitled " **Estimation of Cost Efficiency of fattening pigs, sows, and piglets using SFA approach analysis: evidence from China** ". Those comments are all valuable and very helpful for revising and improving our paper, as well as the important guiding significance to our research.

We have carefully studied the comments of editor and reviewer comments, and based on the comments, the paper has been modified as follows (the red font below is the comments of editor and reviewers, and the black font below is the modification description):

**Responses to the comments of the editors**

**Comment-1:** Please ensure that your manuscript meets PLOS ONE's style requirements, including those for file naming.

**Response:** Thank you sincerely for this comment. Based on the comment of the editor, we modify strictly according to the style requirements of PLOS ONE.

**Comment-2:** If you would like to make changes to your financial disclosure, please include your updated statement in your cover letter. Please state what role the funders took in the study. If the funders had no role, please state: "The funders had no role in study design, data collection and analysis, decision to publish, or preparation of the manuscript."

If this statement is not correct you must amend it as needed.

Please include this amended Role of Funder statement in your cover letter; we will change the online submission form on your behalf.

**Response:** Thank you sincerely for this comment. Based on the comment of the editor, our cover letter includes the updated financial statement. Details are as follows: 'This research is supported by the National Natural Science Foundation of China (grant numbers 71673273); the Natural Science Foundation of Heilongjiang Province China (grant number LH2019G002); and the Humanities and Social Science Foundation of Ministry of education of China (grant number 21YJA790053). The funders had no role in study design, data collection and analysis, decision to publish, or preparation of the manuscript. '

**Comment-3:** We note that Figures 1, 3, 5, and 7 in your submission contain [map/satellite] images which may be copyrighted. We require you to either (1) present written permission from the copyright holder to publish these figures specifically under the CC BY 4.0 license, or (2) remove the figures from your submission.

**Response:** Thank you sincerely for this comment. Based on the comment of the editor, we have removed map-related figures from the revised manuscript. The reason is that the revised manuscript, according to the comments of reviewer # 1, using one stage estimation through SFA, re-estimated the cost efficiency. Since the cost efficiency estimates have changed, we need to draw new maps. The new maps were completed on November 20, 2021. Maps from China Ministry of Natural Resources Standard Map Service System (http://bzdt.ch.mnr.gov.cn/index.html). The standard maps need to be submitted to the natural resources department for review before being used publicly, with a review period of at least 20 days. In this paper, removed maps do not have a substantial impact on the expression of the research content, and all information can still be expressed through tables. Therefore, considering the timeliness of the revised manuscript and the role of maps in the paper, we decided to remove the map information in the revised manuscript. Figure 1, 3, 5, and 7 of the original manuscript are also from the China Ministry of Natural Resources Standard Map Service System (http://bzdt.ch.mnr.gov.cn/index.html).

**Comment-4:** Please include captions for your Supporting Information files at the end of your manuscript, and update any in-text citations to match accordingly.

**Response:** Thank you sincerely for this comment. Based on the comment of the editor, this revised manuscript includes captions for my Supporting Information files. Details are as follows: S1 Table. Input and output indicators for fattening pigs in China for 2008-2019.xlsx

S2 Table. Input and output indicators for sows in China for 2008-2019.xlsx

S3 Table. Input and output indicators for piglets in China for 2008-2019.xlsx

**Comment-5:** In order to improve the quality of your manuscript, I suggest making the improvements indicated by the reviewers, in the same way, I ask you to consider the suggested references.

**Response:** Thank you sincerely for this comment. Based on the comment of the editor, we have improved manuscripts in strict accordance with the opinions of reviewers. In the choice of research methods, this paper refers to the comments given by reviewer #1, combined with the existing literature, considering the applicability of the model, and finally choose to use one stage estimation through SFA. Thank you very much for your references, so that we have a deeper understanding of DEA and Tobit methods.

**Responses to the comments of Reviewer #1**

**Comment:** The paper suffers from a major methodological flaw that precludes its publication in its current form. The present paper uses a two-stage equations to (1) estimate the cost-efficiency through SFA and (2) to analyse the efficiency determinants. This approach is considered to be not the appropriate method in the SFA framework as the error terms of the two equations could be correlated.
 **Response:** Thank you sincerely for this comment, so that this paper has a deeper understanding of the research framework of SFA. Based on the comment of the reviewer, the paper uses the **one-stage estimation through SFA, and the model clearly describes the determinants of inefficiency.** We will describe the changes from three aspects: 1) the form of the model after modification; 2) the basis of the model modification; 3) comparison of results before and after method modification.

**The form of the model after modification**

**The specific form of the Translog-SFA model is as follows:** (For details, please see the manuscript):

$\text{ln}\text{C}_{\text{hit}}\text{=}\text{α}_{\text{h0}}\text{+}\text{α}_{\text{h1}}\text{lny}_{\text{hit}}\text{+}\frac{\text{1}}{\text{2}}\text{α}_{\text{h2}}\left( \text{lny}_{\text{hit}} \right)^{\text{2}}\text{+}\sum_{\text{j=1}}^{\text{3}} \text{β}_{\text{hj}}\text{lnw}_{\text{hjit}}\text{+}\frac{\text{1}}{\text{2}}\sum_{\text{j=1}}^{\text{3}} \sum_{\text{k=1}}^{\text{3}} \text{λ}_{\text{hjk}}\text{lnw}_{\text{hjit}}\text{lnw}_{\text{hkit}}\text{ }$

$\text{ +}\sum_{\text{j=1}}^{\text{3}} \text{θ}_{\text{hj}}\text{lny}_{\text{hit}}\text{lnw}_{\text{hjit}}\text{+}\text{ν}_{\text{hit}}\text{+}\text{μ}_{\text{hit}}\text{+}\text{α}_{\text{h3}}\text{t+}\frac{\text{1}}{\text{2}}\text{α}_{\text{h4}}\text{t}^{\text{2}}$	(1)

Where the subscript $h$ represents the three stages of pig production, $h=1$ represents fattening pigs, $h=2$represents sows, $h=3$ represents piglets; the subscript $i$ represents the province in the sample $\left( i=1,2,\ldots,n \right);$ the subscript $t$ represents year $(t=1,2,\ldots,n)$; $C_{\mathrm{hit}}$is the total cost of $h$ production of province $i$at year t. $y_{\mathrm{hit}}$ is the output of $h$ production of province $i$at year t. $\omega_{\mathrm{hjit}}(j=1,2,\ldots,n)$ is a $j$^th^ input price of $h$ production of province $i$at year t. $\alpha,\beta,\lambda,\theta$are parameters to be estimated; $v_{\mathrm{hit}}$ is a random error term and follows $\text{N}\left( 0, \sigma_{V}^{2} \right)$ ; $\mu_{\mathrm{hit}}$is the cost inefficiency item of $h$ production of province $i$at year t.

Inefficiency effects are independently distributed and $\mu_{\mathrm{hit}}$ arises by truncation (at zero) of the normal distribution with mean, $m_{\mathrm{hit}}$, and variance, $\sigma_{\mu}^{2}$, where $\mu_{\mathrm{hit}}$ is defined by

$\mu_{\mathrm{hit}}=\delta_{h0}+\sum\delta_{h1}z_{\mathrm{hit}}+\varepsilon_{\mathrm{hit}}$	(2)

Through the existing literature review, there are some differences in the influencing factors of cost inefficiency of fattening pigs, sows, and piglets, so this paper constructs the cost inefficiency model of fattening pigs (Formula 3) and the cost inefficiency model of sows and piglets (Formula 4), respectively.

**The specific form of cost inefficiency model can be defined as follows:**

$\mu_{\mathrm{hit}}=\delta_{h0}+\delta_{h1}\mathrm{lnenvi}_{\mathrm{it}}+\delta_{h2}\left( \mathrm{lnenvi}_{\mathrm{it}} \right)^{2}+\delta_{h3}\left( \mathrm{lnenvi}_{\mathrm{it}} \right)^{3}+\delta_{h4}\mathrm{tech}_{\mathrm{it}}+\delta_{h5}\mathrm{disea}_{\mathrm{it}}+\delta_{h6}\mathrm{stru}_{\mathrm{it}}+\delta_{h7}\mathrm{corn}_{\mathrm{it}}+\varepsilon_{\mathrm{hit}}$	（3）

$\mu_{\mathrm{hit}}=\delta_{h0}+\delta_{h1}\mathrm{lnenvi}_{\mathrm{it}}+\delta_{h2}\left( \mathrm{lnenvi}_{\mathrm{it}} \right)^{2}+\delta_{h3}\left( \mathrm{lnenvi}_{\mathrm{it}} \right)^{3}+\delta_{h4}\mathrm{tech}_{\mathrm{it}}+\delta_{h5}\mathrm{disea}_{\mathrm{it}}+\delta_{h6}\mathrm{stru}_{\mathrm{it}}+\delta_{h7}p\mathrm{pig}_{\mathrm{it}}+\varepsilon_{\mathrm{hit}}$	（4）

Where $\mathrm{lnenvi}_{\mathrm{it}}$is the intensity of environmental regulation of the $i$ province at year $t$. It is mainly evaluated from four aspects: policy intensity, policy objectives, policy measures, and policy feedback. Testing the nonlinear relationship between environmental regulation and cost efficiency by quadratic and cubic of environmental regulation intensity. $\mathrm{disea}_{\mathrm{it}}$is the major epidemic intensity of the hog industry of the $i$ province at year $t$, which is equal to the number of deaths of major diseases of fattening pigs and the number of compulsory culling divided by the total number of fattening pigs. $\mathrm{tech}_{\mathrm{it}}$is the intensity of technology input of the $i$ Province at year $t$, which is equal to the ratio of R & D input to GDP; $\mathrm{stru}_{\mathrm{it}}$ is the industrial structure of the $i$ province at year $t$, which is equal to the ratio of primary industry output value to GDP. It is used to represent the agricultural resource endowment of the region. $\mathrm{corn}_{\mathrm{it}}$ is the corn feed price of the $i$ province at year $t$ to reflect the impact of factor markets prices on the efficiency of the hog industry. $\mathrm{ppig}_{\mathrm{it}}$ is the pig price of the $i$ province at year $t$ to reflect the impact of pig price on the cost efficiency of sows and piglets.

**The basis of the model modification**

According to the reviewers' comments, this paper uses SFA to estimate cost efficiency and analyze the influencing factors of inefficiency. Cobb-Douglas cost function and Translog cost function are the two most widely used stochastic frontier cost functions. Because the latter has the characteristics of variable elasticity of substitution of input factors, it is closer to the actual process of variable scale income of pig breeding. Therefore, this study uses the latter to estimate cost efficiency. This paper refers to the literature in Table 1 to set the Translog cost function.

**Table 1. The literature basis of Translog cost function setting**

| **Tittle** | **Author** | **Journal** |
| --- | --- | --- |
| US all-cargo carriers' cost structure and efficiency: A stochastic frontier analysis | Balliauw,  Matteo,  Meersman, et al | Transportation research, Part A. Policy and practice |
| Estimating hospital inefficiency: does case mix matter? | Rosko M,  Chilingerian J | Journal of Medical Systems |
| Cost efficiency and the effect of mergers on the Taiwanese banking industry | Peng,Ya-Hui  Wang, Kehluh | The Service Industries Journal |
| A bioeconomic approach to optimize mussel culture production. | Isabel Fuentes-Santos  Alhambra M. Cubillo  U. Labarta | Reviews in Aquaculture |
| Cost and technical efficiency of German hospitals: does ownership matter? | Herr, Annika | Health economics |
| Using stochastic frontier analysis to measure the impact of weather on the efficiency of electricity distribution businesses in developing economies | Anaya, Karim L. Michael G. Pollitt | Eur. J. Oper. Res |
| Technical change and productivity growth in airports: A case study | Barros, Carlos Pestana | Transportation Research Part A-policy and Practice |

To ensure the rationality of index selection, this paper sorts out the theory of index construction (Fig.1) and determines the specific research variables by referring to the existing literature (For details, please see the manuscript (lines 127-155, 170-189)).

**Fig.1 Indicator selection basis**

**Comparison of results before and after method modification**.

According to the reviewer's comment, the research method is changed from using ‘a two-stage equation’ to ‘one stage estimation through SFA’. We will describe the differences between the original manuscript and the revised manuscript from four aspects: research object, research time, research methods, and research results（Table 2）. This revised manuscript adds research objects and considers the latest published data. The validity of the model is improved compared with the original manuscript. The conclusions of the revised manuscript are richer than the old draft. It is worth mentioning that the revised manuscript tests the nonlinear relationship between environmental regulation and cost efficiency by quadratic and cubic environmental regulation intensity. Once again, we would like to express our sincere thanks to the reviewer for your comments on revising the paper. We hope the revised manuscript is suitable for the journal’s desired standard.

**Table2	Comparison of results before and after method modification**

| Manuscript  Difference | | **Original manuscript** | **Revised manuscript** |
| --- | --- | --- | --- |
| *Research object* | | 23 pig-breeding provinces | 29 pig-breeding provinces |
| *Research time* | | 2008-2018 | 2008-2019 |
| *Research methods* |  | SFA+Tobit | SFA |
| The number of significant parameters in SFA model | Fattening pigs | 11 | 20 |
|  | Sows | 12 | 20 |
|  | Piglets | 14 | 21 |
| *Research results* |  |  |  |
| Cost efficiency estimates | Fattening pigs | 0.91 | 0.77 |
|  | Sows | 0.78 | 0.79 |
|  | Piglets | 0.82 | 0.53 |
| Cost efficiency estimates by region | Fattening pigs | The highest in the central region | The highest in the northeast region |
|  | Sows | The highest in the central region | The highest in the central region |
|  | Piglets | The highest in the central region | The highest in the northeast region |
| The impact of environmental regulation on cost efficiency | Fattening pigs | Negative impact | 'N '-shaped relationship |
|  | Sows | Negative impact | inverted ' N '-shaped relationship |
|  | Piglets | Negative impact | inverted ' N '-shaped relationship |

**Responses to the comments of Reviewer #2**

**Comment-1:** Introduction is well written and characterize clearly the practical importance of research. However, there is no clearly defined aim of the study at the end of Introduction. The last paragraph seem to be more M&M summary, so the aim of the study is defined only as one general sentence at the beginning of Abstract. It is not enough, and clear definition of the aim of the study must be add.

**Response:** Thank the reviewer for your approval and comments. Based on the comment of the reviewer, the aim of the study is clearly defined at the end of the introduction.

This paper aims to evaluate the efficiency of fattening pigs, sows, and piglets, and focus on the impact of environmental regulation on cost efficiency. This paper hopes to provide experience for promoting the improvement of pig breeding efficiency or avoiding the loss of pig breeding efficiency under the dual constraints of resources and environment.

**Comment-2:** Additionally, I would suggest to add information about more international currency (e.g. USD), because the value of yuan is difficult to interpret for international reader.

**Response:** Thank the reviewer for your comments, so that the paper can be more convenient for international readers to interpret. Based on the comment of the reviewer, this paper adds the information of international currency (USD) from two aspects. First, for the expression of price information in the introduction, which is described in USD. For example, this paper changes the previous ' 1930.41 yuan/head, 535.85 yuan/head, and 1654.6 yuan/head ' to ' 301.9USD/head, 83.8USD/head, and 258.8USD/head’. Second, for the original manuscript of ' Table 3. Statistical characteristics of the main variables cited in the stochastic frontier cost model ', this paper adds the information of international currency (USD). Specific changes can be seen in table 3. The data accounting unit in the parentheses in Table 3 is USD.

**Table 3.**  **Descriptive statistical characteristics of main variables**

| Variable | Explanations | Unit | Mean | Standard deviation | Minimum | Maximum |
| --- | --- | --- | --- | --- | --- | --- |
| $C_{1}$ | Total cost of fattening pigs | CNY(USD)^a^ | 1417.794(221.7431) | 196.1859 (30.6835) | 993.2660 (155.3468) | 2015.8570 (315.2800) |
| $C_{2}$ | Total cost of sows | CNY(USD) | 4341.278(678.9758) | 791.9975 (123.8684) | 2586.6000(404.5443) | 8848.4920 (1383.9040) |
| $C_{3}$ | Total cost of piglets | CNY(USD) | 403.9777(63.18211) | 106.5857 (16.6700) | 176.7559(27.6446) | 777.9240 (121.6673) |
| $y_{1}$ | The slaughter weight of fattening pigs | Kg | 116.4690 | 9.3288 | 98.3107 | 154.9701 |
| $y_{2}$ | Pigs weaned per Sow per Year | Head | 25.8377 | 4.9127 | 10.3012 | 39.2397 |
| $y_{3}$ | Market pigs per Sowper Year | Head | 22.6855 | 4.3133 | 9.0444 | 34.4525 |
| $\omega_{11}$ | Feed price | CNY/Kg (USD/Kg) | 2.4282(0.3798) | 0.4921 (0.0770) | 1.3642 (0.2134) | 5.2329 (0.8184) |
| $\omega_{12}$ | Price of piglet | CNY/Kg (USD/Kg) | 24.6172(3.8501) | 7.6423 (1.1953) | 10.5970 (1.6574) | 48.0359 (7.5128) |
| $\omega_{13}$ | Price of labor force | CNY/ Day (USD/ Day) | 54.7365(8.5608) | 18.8068(2.9414) | 21.6094(3.3797) | 100.2358 (15.6769) |
| $\omega_{22}$ | Price of replacement gilt | CNY/Kg (USD/Kg) | 11.6552(1.8229) | 2.3619 (0.3694) | 6.5480 (1.0241) | 25.1181 (3.9285) |
| $\omega_{23}$ | Daily labor price | CNY/Day (USD/Day) | 47.7061(7.4612) | 20.2367(3.1650) | 11.8000 (1.8455) | 104.3760 (16.3244) |
| $\omega_{32}$ | Sow apportionment price | CNY/Kg (USD/Kg) | 174.3332(27.2657) | 46.1747 (7.2217) | 93.2126(14.5785) | 345.8629 (54.0930) |
| $\mathrm{envi}$ | Environmental regulation intensity | - | 1827.7440 | 668.3808 | 390.0000 | 3829.0000 |
| $\mathrm{tech}$ | Technology input intensity | - | 1.4270 | 0.7771 | 0.2200 | 4.6800 |
| disea | Pig disease intensity | - | 0.0002 | 0.0010 | 0.0000 | 0.0120 |
| $\mathrm{corn}$ | Corn feed price | CNY/Kg (USD/Kg) | 1.8849(0.2948) | 0.2835 (0.0443) | 1.2194 (0.1907) | 2.5407 (0.3974) |
| $\mathrm{stru}$ | Industrial structure | - | 10.5032 | 5.1453 | 0.3000 | 30.0000 |
| $\mathrm{ppig}$ | Pig market price | CNY/Kg (USD/Kg) | 13.3537(2.0885) | 2.0212 (0.3161) | 9.0854(1.4210) | 18.8935 (2.9549) |

Note: The sample size of fattening pigs, sows, and piglets is 253.

^a^ This paper uses the exchange rate to convert the unit of price variables from CNY to USD. The data accounting unit in parentheses in the above table is USD.

The exchange rate is: 1CNY=0.1564$ (Statistical time 2021-11-12 16: 13)

**Comment-3:** The study is based on retrospective analysis of macroscale data, that is why the most important is to define clearly variables to analyze and models. The models are defined, however variables only generally in the text, and a little more detailed in table 3. This table must be corrected, because variables in table poorly correspond to the text of M&M, and thus the table is difficult to interpret. First of all, variables in the table should be named to be interpretable reading only table (without text). And second, variables should be characterized also with units (is this only cost in currency, or the other units were used).

**Response:** Thank the reviewer very much for your comments, which not only enables this paper to define variables more clearly but also provides great inspiration for our future paper writing. Based on the comment of the reviewer, first, this paper explains the meaning of variables, so that the variables can be an interpretable reading only table. Second, this paper adds units of variables. For currency variables, this paper uses two units of CNY and USD to describe. Specific modifications are shown in table 3.

**Comment-4:** Results are described very detailed, and after corrections in M&M should be OK. Discussion is very general and seem to be more reediting of results than critical interpretation. The specificity of data analysis suggests, that maybe it would be better to compose manuscript without separate discussion, and change it to Results and discussion as one section. Anyway, this section should and with conclusion. There is no such separate section, and the manuscript seem to have no clear end, and no suggestion for practice. This must be corrected and completed**.**

**Response:** Thank the reviewer very much for your comments, so that this paper can be improved in structure and content arrangement. Based on the comment of the reviewer, this paper composes the results and discussion as a section and adds the section of conclusion in the revised manuscript. The conclusion includes two parts. One is the main conclusion of this paper, and the other is suggestions for practice (For details, please see the manuscript (lines 569-605)). Table 4 and Table 5 express the content of the conclusion part in the revised manuscript in the form of tables, hoping to facilitate the reviewer's understanding and save your time.

**Table 4. The main conclusion of this paper**

|  | **Fattening pigs** | **Sows** | **Piglets** |
| --- | --- | --- | --- |
| **Cost efficiency estimates** | Based on these results, by operating at the efficient frontier the sample fattening pigs producers would be able to reduce their production cost by 23%. | Based on these results, by operating at the efficient frontier the sample fattening pigs producers would be able to reduce their production cost by 21%. | Based on these results, by operating at the efficient frontier the sample fattening pigs producers would be able to reduce their production cost by 47% |
| **The impact of environmental regulation on cost efficiency** | Further improving the intensity of environmental regulation can achieve the dual goals of environmental governance and cost reduction. | Appropriately reducing the intensity of environmental regulation can avoid cost efficiency estimates losses. | Environmental regulation does not form an effective incentive for cost efficiency |
| **The impact of other influencing factors on cost efficiency** | Increasing the intensity of technology input can also achieve efficiency gains | Farmers improve the intensity of technology input and transfer to areas with high agricultural factor resource endowment can achieve cost efficiency gains. | Farmers to reduce the intensity of technology input, establish price early warning mechanism can avoid cost efficiency estimates loss. |

**Table 5. Suggestions for practice**

|  | | **Suggestions** |
| --- | --- | --- |
| Farmers | 1. To avoid the loss of efficiency, it is necessary for farmers to actively adapt to the adjustment of environmental regulation policies through organic fertilizer processing and biogas project construction. 2. To achieve the gain of cost efficiency, it is necessary for fattening pigs and sows breeding enterprises to increase the level of technological R&D investment, strengthen the dissemination and training of technological achievements, and improve the professional quality of breeding practitioners 3. To avoid market risks and efficiency losses, farmers can make full use of ' insurance + futures ' and other emerging financial instruments | |
| Hog industry | 1. The development of the hog industry in the northeast region should focus on fattening pigs and piglets, and the development of the hog industry in the central region should focus on sows. 2. To achieve the gain of cost efficiency, it is necessary for fattening pigs and sows breeding enterprises to increase the level of technological R&D investment, strengthen the dissemination and training of technological achievements, and improve the professional quality of breeding practitioners. 3. the hog breeding industry should establish a price early warning mechanism, monitor the price information and supply and demand status of each link of the industrial chain in all aspects. In addition, timely disclosure information to avoid panic among farmers caused by external shocks such as major animal epidemics. | |

**Comment-5:** To summarize, in my opinion the manuscript could be interesting, but must be improved. Minor language revision is also required.

**Response:** Thank the reviewer for your comments. With full reference to reviewer opinions, to improve the English level of the manuscript, we invited native speakers to check the grammar, style, and syntax of the manuscript to polish the English and pay attention to the expression paradigm. For the professional vocabulary involved in the paper, this paper selects the most frequently used expression as the expression of this vocabulary according to the number of times they appear in published English papers.

With best regards

Yours sincerely,

Gangyi Wang

Chang’e Zhao

Yuzhuo Shen

Ni Yin
